# Do mixed-species groups of capuchin (*Sapajus apella*) and squirrel monkeys (*Saimiri sciureus*) synchronize their behaviour?

**DOI:** 10.1098/rstb.2022.0111

**Published:** 2023-06-05

**Authors:** Sophia Daoudi-Simison, Eoin O'Sullivan, Genevieve Moat, Phyllis C. Lee, Hannah M. Buchanan-Smith

**Affiliations:** ^1^ School of Psychology, Faculty of Medical Sciences, Newcastle University, Newcastle upon Tyne NE2 4DR, UK; ^2^ Psychology, Faculty of Natural Sciences, University of Stirling, Stirling FK9 4LA, UK; ^3^ School of Psychology and Neuroscience, University of St Andrews, Fife KY16 9AJ, UK; ^4^ School of Computing, Faculty of Science, Agriculture and Engineering, Newcastle University, Newcastle upon Tyne NE1 7RU, UK

**Keywords:** capuchin, squirrel monkey, mixed-species group, behaviour, synchrony

## Abstract

In the wild, coordinated behaviour across group members is essential for maintaining spatial coherence, with potential implications for individual fitness. Such coordination often leads to behavioural synchrony (performing the same behaviour at the same time). Tufted capuchins (*Sapajus apella*) and squirrel monkeys (*Saimiri sciureus*) are known to form mixed-species groups (MSGs), travelling and foraging together. Yet, it is unclear if it is necessary to synchronize behaviours in captivity when ecological pressures are minimal compared to the wild. We investigated the extent to which two MSGs of capuchins (*N =* 35) and squirrel monkeys (*N* = 26) synchronized their behaviour with conspecifics and heterospecifics at the Living Links to Human Evolution Research Centre, RZSS, Edinburgh Zoo, UK. Group activities were sampled by instantaneous scans of all visible individuals. Scans (*n* = 180) were analysed for five most frequently observed behaviours. Intraspecies synchrony was calculated using Simpson's Diversity Index, and interspecies synchrony was measured using cross-correlations. Intraspecific synchrony was significantly greater compared to randomly aggregated data, while cross-correlations indicated interspecific asynchrony. Living together did not lead to interspecific synchrony as may be expected given the coordination and behaviour described in the wild, and shared husbandry in captivity. Overall, our findings highlight differences in the behavioural structure of single- versus MSGs.

This article is part of the theme issue ‘Mixed-species groups and aggregations: shaping ecological and behavioural patterns and processes’.

## 1. Background

Synchronized behaviour is an important feature of animal groups and occurs when individuals perform the same activity while in the same location. Furthermore, species that live sympatrically and aggregate around shared resources are likely (at least part of the time) to synchronize their behaviours. This behavioural synchrony (hereafter synchrony) is arguably a contributing factor of the *double benefits of grouping hypothesis*, where foraging efficiency increases and the risk of predation decreases for individuals in groups [1,2]. Furthermore, theoretical studies have demonstrated that group cohesion is maintained by the synchronous activity of group members [3,4] because it cannot be maintained if some engage in stationary activities (e.g. grooming, resting) while others engage in movement activities (e.g. foraging, locomotion, playing). Thus, both ecological and social mechanisms are considered to drive synchrony, including social facilitation, copying conspecifics and the selfish herd [1,5,6]. However, little research has explicitly tested between competing hypotheses.

Group-level synchrony remains an understudied area within the non-human primate literature, with much previous research focussing on either coordinated action within dyads [7], collective behaviour [8] or response to specific natural phenomena such as synchronized births [9]. One main difficulty is determining the extent of synchrony when observing more than two animals [10]. For instance, while an individual may be behaving in synchrony with some members of its group, it will be asynchronous with others. Determinants of synchrony can be based on age, sex, physiological and morphological characteristics, hierarchical position or even the presence of another species [11,12]. When a ‘group’ consists of more than one species an additional layer of complexity is added. Thus, despite the interest ethologists have shown in the study of mixed-species groups (MSGs), there have been few attempts to examine their synchrony as a measure/indicator of ‘groupness' [13]. Simultaneous responses to alarm calls [14] and synchronized foraging [15] have been observed (at the group level) in mixed-species bird flocks, but there is as yet no work on similar phenomena in mixed-species primate groups.

Capuchins (*Sapajus* sp.) and squirrel monkeys (*Saimiri* sp.) are sympatric across much of South America and they are two of the most commonly reported Platyrrhine primates to form MSGs [16–21]. Squirrel monkeys are considerably smaller than capuchins, weighing approximately 0.60–1.20 kg compared to 1.30–4.80 kg [22], which is a larger difference than seen in other primate MSGs [21,23,24]. While no data are available to specifically answer whether capuchins and squirrel monkeys are synchronized in the wild, observational research has found that these groups often perform similar behaviours in the same locations [16–19,21]. They have been frequently observed travelling and foraging together [21], with both species typically engaging in more foraging and less vigilant behaviour in the presence of the other than when in single-species groups (i.e. *double benefits hypothesis* [2]). Squirrel monkeys are able to take advantage of the extractive foraging techniques of capuchins, and during periods of food scarcity, capuchins are able to benefit from the squirrel monkeys' knowledge of multiple food sources [21]. In addition to foraging benefits, squirrel monkeys also benefit from capuchin alarm calls; indeed, they have been observed to respond more readily to capuchin alarm calls than to those of conspecifics [21].

As synchrony is difficult to examine using classical analyses based on the probability distribution function, here we implemented permutation tests used by previous studies of within- and between-species synchrony [25,26] to ask whether capuchin and squirrel monkeys synchronize their behaviour in captive settings when housed in MSGs. Although captive settings differ from the wild, they provide a more controlled environment to study synchrony and help test the predictions of the *double benefits hypothesis*, as well as the role of the current environmental conditions on synchrony. While synchrony in captivity could at least in part be based on circadian rhythms [27,28] and/or daily shared husbandry routines (e.g. timing of feeds may lead to synchronized foraging), the evolutionary history between these two species forming MSGs in the wild may also facilitate synchrony in captivity due to social mechanisms [16–19,21,29]. Thus, this method of analysis helps us to quantify the ‘groupness' of the monkeys in terms of synchrony.

Based on their natural association in the wild and shared husbandry routines in captivity, we expected capuchin and squirrel monkeys to synchronize their behaviours (i.e. as a ‘true’ MSG). Evidence of these species synchronizing specific behaviours would shed light on the benefits of mixed-species membership. Given the paucity of research on within-species behavioural synchrony, we also explored for the first time whether individual groups of capuchin and squirrel monkeys synchronized their behaviour.

## 2. Methods

### (a) Subjects

Study animals were two separate groups (west and east) of capuchin and squirrel monkeys at the Living Links to Human Evolution Research Centre, Royal Zoological Society Scotland, Edinburgh Zoo (hereafter Living Links). Both groups of capuchins contained adults, subadults and juveniles of both sexes, whereas both squirrel monkey groups contained only one adult male, with the rest being a mix of adult and sub-adult (one juvenile in the west) females. There were 17 individuals for both capuchin and squirrel monkey groups in the east exhibit, 18 individuals in the west capuchin and nine squirrel monkeys. Each monkey could be individually identified by their facial characteristics and additional artificial aids for squirrel monkeys (chain collars with colour coded beads).

### (b) Housing and husbandry

**Figure 1.**
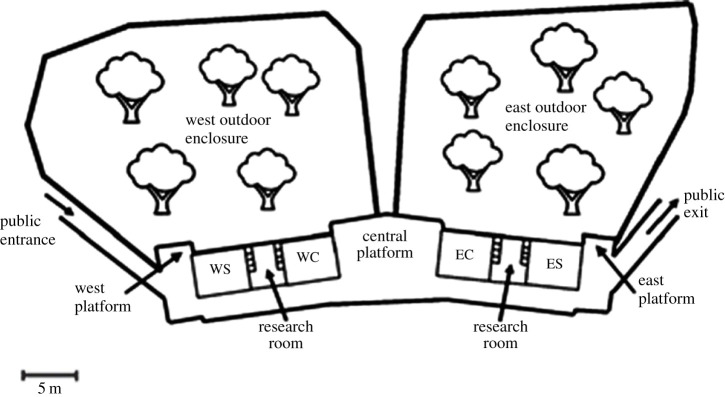
Schematic diagram (approximately to scale) of Living Links (adapted from Leonardi *et al*. [32]). The shared outside enclosures are shown for the east and west groups, along with the monkeys’ indoor enclosures (WS and WC = west squirrel monkeys and west capuchins; ES and EC = east squirrel monkeys and east capuchins) and research rooms, and the indoor and outdoor public viewing areas.

Living Links was specifically designed to accommodate both species of monkey and provides spacious naturalistic enclosures to allow them to exhibit species-typical behaviours, promoting high welfare [30,31]. Both the west and east exhibits include an indoor enclosure for capuchins (7 m × 4.5 m × 6 m high) and an indoor enclosure for squirrel monkeys (5.5 m × 4.5 m × 6 m high). Squirrel monkeys can enter the capuchin indoor enclosure but not *vice versa*. When the monkeys are indoors they have the potential to see each other in the research rooms, as well as see outdoors through large windows. The shared outdoor enclosures are approximately 900 m^2^, with the west and east groups being physically separated by a double fence line. They are however still able to communicate vocally and visually (e.g. when high up in the trees; figure 1).

### (c) Design and procedure

Data were collected during June–August 2015. All observable monkeys (both capuchin and squirrel monkeys) were sampled in each scan, with the exception of the west squirrel monkey infants that were born later in the study (August 2015). Scans were collected for all members of either west or east groups, across enclosures within a maximum of 15 min intervals [32]. We recorded each individual in the indoor enclosures first, choosing individuals from left to right, then individuals in the outdoor enclosure. If an individual was not observed during a scan, then that individual would be recorded as out-of-sight. Observations were taken from one of the three public viewing platforms: the west platform, the central platform or the east platform, or the indoor corridor [32]. Recording took place throughout the week (Monday–Sunday) between 09.00 and 17.00, accumulating a total of 180 scans divided equally between the west and east groups. An average of 9.47 scans were made on any day (min = 4; max = 14; *n* = 19 days). We avoided collecting data during cubicle research testing times (Mondays, Tuesdays, Thursdays and Fridays: 11.15–12.45 and 14.15–15.45) so that all individuals had the potential to be sampled. During scans a monkey could be inside or outside, and the activity of each individual was recorded. The east groups were co-present in enclosures for 84% of scans, while co-occurrence was only 34% for the west (electronic supplementary material, table S1).

For the purpose of the statistical analysis, we compiled the west and east groups into one dataset. That is, synchrony scores for a given observation period were calculated separately for each of the two groups of capuchin and squirrel monkeys (i.e. west and east). However, we then aggregated the scores from west and east groups to test our general hypotheses regarding synchrony in each species. Similarly, when comparing synchrony across species, correlations were computed separately for west and east groups, and these correlations were then aggregated to test our hypothesis. Scores were aggregated to present an overall picture of synchrony at the single- and mixed-species level; however, our analyses were also computed separately for each group and are reported in electronic supplementary material. The behaviours of vigilance, foraging, locomotion, resting and playing (table 1 for definitions) were selected for analysis, given that they are unambiguous to observers, occurred at sufficient frequency and have been reported in other studies on these species [32,33]. All data were collected by a research assistant and inter-observer reliability (IOR) followed Buchanan-Smith *et al*. [34] (see electronic supplementary material for details).

**Table 1.** Definition of the five behavioural categories most frequently recorded, adapted from Leonardi *et al*. [32].

behaviour	definition
Vigilant (V)	Sitting or standing, with eyes actively scanning the surroundings.
Foraging (F)	Searching for food, including ground digging, scanning the environment for insects or pieces of food, and eating.
Locomotion (L)	Monkey is moving in relation to its surroundings: slow locomotion—moving, usually walking, with no jumping or running; agitated/fast locomotion—movements are made at a rapid pace, that is, at a running speed, and also include jumping and leaping when there is more than one leap/jump made, that is, a succession. Is not scored when playing.
Resting (R)	Sleeping or in a state of calmness, with the body relaxed in a stationary position. Eyes may be closed or open, but not actively scanning the environment. Also includes grooming for capuchins.
Playing (P)	Engages in high activity interaction (e.g. chase, rough and tumble, mock wrestling) with other individuals. This can include non-aggressive physical contact, or occur at a distance, for example, hopping and running, steep leaps (almost vertical jumps with minimal forward locomotion) or swinging by the feet, while visually checking/coordinating with play partners. It is inherently synchronized.

### (d) Data analysis

We calculated behavioural synchrony (*BS*) for each scan using a simple index that measures diversity in categorical data, as described by King & Cowlishaw [25]—Simpson's diversity index (SDI):

$$BS=\;\overset{C}{\underset{i=1}{\sum }}\frac{n_{i}\;(n_{i}\;\text{–}\;1)}{N\;(N\;\text{–}\;1)}$$

where *n_i_* is the total number of observable individuals engaged in specific behaviour *i*, i∈[1,2,3,4,5], *C* is the total number of observed behaviours and *N* is the total number of individuals in view. Synchrony scores near zero indicate asynchrony, while scores closer to one indicate more homogeneity and therefore better synchrony.

To examine within-species synchrony data, we first computed the sampling weights for each species (capuchins: 0.468, 0.234, 0.118, 0.106, 0.073; squirrel monkeys: 0.608, 0.152, 0.126, 0.111, 0.002), which was based on the proportion of each of the five behaviours (vigilant, foraging, locomotion, resting and playing) present in the 180 scans. Using these sampling weights, we generated data such that a random sample was drawn based on the sampling weights, with the total number of observations of each scan remaining [25]. We ran 1000 iterations such that 180 scans would be generated for one iteration, and the average of the synchrony scores of the generated scans was computed.

Permutation tests are recommended in cases where observations are not independent (e.g. observations of behaviours gathered on the same morning will likely be correlated) and essentially allow comparison of observed patterns in a dataset with patterns obtained from resampling of the same data. (For a more detailed discussion of the benefit of permutation tests for the study of synchrony see [26].) If the observed pattern of within-group synchrony is greater than values collected from randomly generated data, we can be confident that animals are synchronizing behaviour more than might be expected due to simple frequencies of behaviours.

To examine synchrony between-species, we performed cross-correlations using Pearson's correlation coefficient to examine correlations between the occurrence of specific behaviours. The examination of correlations between specific behaviours allows a more detailed analysis of specific mixed-species synchrony patterns, in comparison to the group-level analysis reported for the single-species data. In accordance with Foerder *et al*. [26], we used a randomization test where we combined the data containing capuchin and squirrel monkeys' behaviours, shuffled the order of the behaviour at random and divided the generated scan into the corresponding numbers of each species. For instance, after combining the data, we may obtain a scan with four capuchins and three squirrel monkeys exhibiting the behaviour A (frequency 4), behaviour B (frequency 2) and behaviour C (frequency 1), respectively. The conduct list will be AAAABBC. After being randomly shuffled, the generated behaviour of the scan could be BAAABCA. Afterwards, we split the generated behaviour list with the corresponding number of each species, such that for capuchins, the randomized behaviour would be BAAA, and for squirrel monkeys the randomized behaviour list would be BCA.

Similar to the within-species synchrony score, the cross-correlations were computed for each randomly shuffled scan and an average cross-validation for each behaviour was calculated based on 180 scans. This process was repeated 1000 times, acquiring a total of 5000 cross-correlations. This approach allows us to compare correlations observed in the actual MSG with samples generated from treating the MSG as one group.

To determine whether our within- and between-species synchrony scores are higher/lower than we might expect by chance, we evaluated the generated data's observed values using *p*-values [35]. The null hypothesis is that the observed value comes from the same distribution as the randomly generated data, and the *p*-value was calculated as the proportion of samples with test statistics larger/smaller than our observed data. For example, if, 90% of the synchrony scores calculated from randomly generated data were lower than the observed synchrony score, our *p*-value would be 0.10.

All statistical tests were performed using the software package R (v.4.1.3) in the RStudio environment ([36,37]—see code here [https://github.com/GenMoat/Behavioural-Synchrony-]), at an *α* of 0.05 [38]. The *p*-value was the proportion of cases in the distribution from the randomized datasets that were equal to or more extreme than the observed measure.

## 3. Results

**Figure 2.**
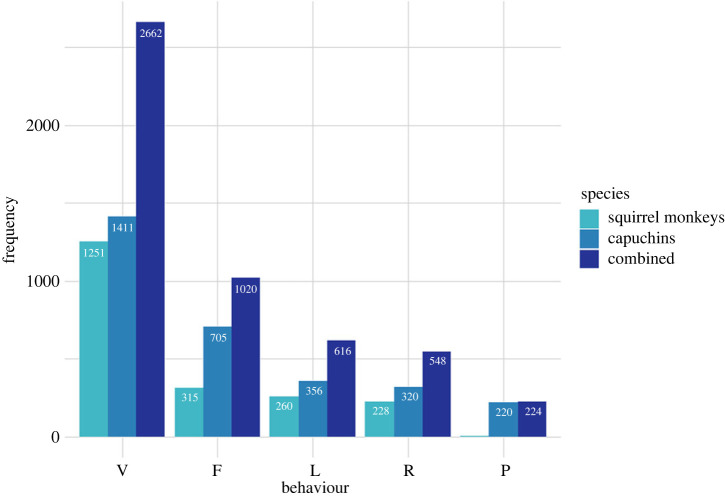
Frequency of records of monkeys (single-species and combined) engaged in each behaviour (V, vigilant; F, foraging; L, locomotion; R, resting; P, playing), based on 180 scans. (Online version in colour.)

For both species, vigilance was the most frequently observed behaviour, followed by foraging, locomotion, resting and playing (figure 2).

**Figure 3.**
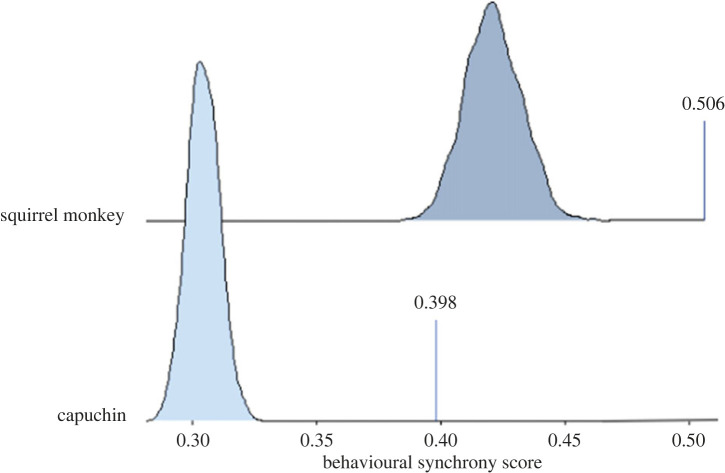
Distributions based on randomization tests (1000 permutations) for squirrel monkeys and capuchins. Vertical lines represent the observed behavioural synchrony score. (Online version in colour.)

For the compiled data, the mean synchrony for 180 scans was 0.395 (s.d. = 0.125), for capuchins 0.398 (s.d. = 0.142), and for squirrel monkeys 0.506 (s.d. = 0.194). When comparing the within-species scores to those generated from 1000 randomly generated datasets, the observed synchrony scores were significantly greater (i.e. *p* < 0.001, figure 3). For capuchins, the grand mean was 0.304 (max = 0.323, min = 0.288). For squirrel monkeys, the grand mean synchrony across 1000 datasets was 0.421 (max = 0.462, min = 0.386). We also examined mean synchrony scores separately for west and east mixed- and single-species groups and found that they were not dissimilar to the compiled dataset, indicating that being co-present in enclosures makes little difference to synchrony (electronic supplementary material, table S4).

**Figure 4.**
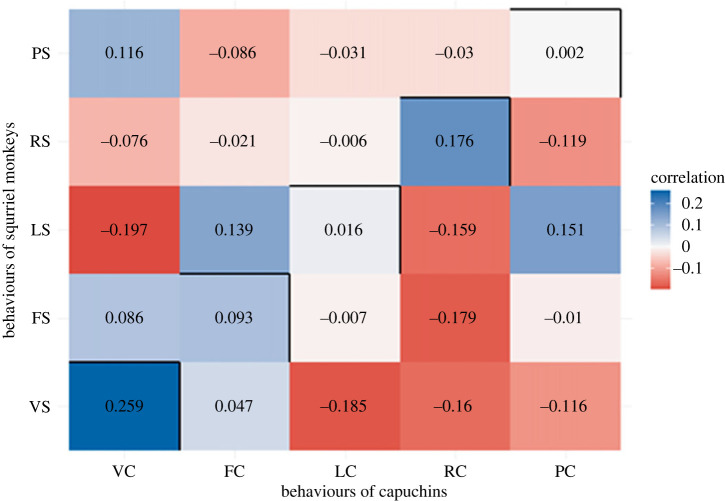
Cross-correlation of the five behaviours (V, vigilant; F, foraging; L, locomotion; R, resting; P, playing) between capuchins (C) and squirrel monkeys (S). Darker colours indicate stronger relationships (see key). (Online version in colour.)

We examined between-species synchrony using Pearson's correlation coefficient between specific behaviours performed by capuchins and squirrel monkeys (figure 4).

To examine whether these relationships were greater/smaller than expected by chance, we compared the observed correlations with the distribution of correlation coefficients calculated from datasets generated from the original data (see table 2 for the observed correlations and descriptive statistics for the randomized datasets). We found that for all five behavioural categories, the observed level of behavioural coordination between-species was significantly less than would be expected based upon the randomization tests, indicating that the monkeys were asynchronous in their behaviours. The *p*-value is the proportion of samples that have a mean cross-correlation value (*r*) that is larger or smaller (or more extreme) than that of our observed data.

**Table 2.** Observed and predicted (based on a mean of 1000 permutations) Pearson correlations between capuchin and squirrel monkeys for each of the five behaviours measured.

	activity				
	vigilance	foraging	locomotion	resting	playing
**observed cross correlation**	0.259**	0.093**	0.016**	0.176**	0.002**
**randomization test distributions of cross-correlations (based on 1000 permutations)**					
mean	0.407	0.487	0.293	0.702	0.425
s.d.	0.040	0.042	0.053	0.035	0.064
max	0.537	0.626	0.465	0.807	0.612
min	0.266	0.355	0.136	0.585	0.201

**p < 0.001, based on randomization test.

## 4. Discussion

We aimed to examine, for the first time, mixed-species synchrony in primates, by adapting a modelling approach used in single-species analysis [25] and studies of mixed-species synchrony in birds [26]. The findings were not consistent with our prediction that if the capuchins and squirrel monkeys were synchronizing their behaviour, the observed correlations would be similar to those found when treating the mixed-group as one group. While mean synchrony scores for the observed data indicated that within-species, capuchins and squirrel monkeys were synchronizing their behaviour, observed cross-correlations in behaviours (between-species) were lower than those generated with randomly assigned groups of comparable sizes.

The methods we have used to analyse synchrony in our data can be applied more generally to any situations in which animals can be classified in terms of their group membership (e.g. by species (if in MSGs), sex, age), and importantly allow computation at the group level rather than individual or dyadic [25,26]. Furthermore, our measure of within-group synchrony (SDI) and a measure of between-group synchrony (cross-correlations) provide a better overall picture of species distinctiveness in MSGs through synchrony than either measure alone.

Previous models suggest that a group can only be spatially coherent if its members synchronize activities [3,4]. Furthermore, individuals similar in body size/mass (and reproductive state) will have similar activity budgets [39,40], and so synchrony can be more readily maintained among them [25,41]. Our focal species have quite a large body size/mass differential compared to some other primate mixed-species combinations [22–24], wild capuchins and squirrel monkeys do share predators (e.g. snakes and birds of prey), and when they are observed in MSGs they are described as coordinating behaviours (i.e. locomotion, increased foraging and decreased vigilance) [16–21]. It can be suggested that those individuals who show the least coordination might be more subject to risks from predators, resulting in local pressure for synchrony. Thus, while it is not conclusive that the behaviours of the two species are synchronized, coordination could be indicative of between-species synchrony.

In captivity, environmental pressures such as predation or food limitation that would typically drive the *double benefits of grouping hypothesis* [2] are largely absent. Unexpectedly, vigilance was the most synchronous of the five behaviours analysed between species (although the relationships were still weak, *r* < 0.3). Typically, vigilant behaviour is lower in larger single species and MSGs in the wild, allowing individuals to focus more on other behaviours such as foraging [42,43]. In captivity, this high level of vigilance could also be due to the differences in size. The smaller squirrel monkeys may still be at risk of predation by raptors when in the outdoor enclosure compared to the larger capuchins [32], and while there are areas that mimic their natural habitat (forest understory, dense foliage and lianas; see [18]) such as evergreen bushes, there is much open space that could be perceived as areas of risk. Furthermore, squirrel monkeys are highly vigilant while foraging for prey due in part to their insectivorous diet [44], while capuchins have a more varied diet [17,22]. Thus, synchrony of group behaviours in captivity could be based on other ecological (e.g. circadian rhythms due to shared husbandry schedules) or social factors (e.g. social zeitgebers). Perhaps a combination of difference in size/mass and phase shifts in circadian rhythms, in addition to sampling methods (i.e. observers were unable to gain an exact snapshot of all behaviours of all individuals) may have contributed to an overall lack of synchrony between the monkeys at Living Links.

The finding that within-species synchrony was significantly greater than expected by chance replicates findings in MSGs of penguins [26] and single-species groups of baboons and macaques [25,45]. However, the squirrel monkeys appeared to be more synchronized than were the capuchins. The smaller total number of squirrel monkeys compared to capuchins could have resulted in a reduced chance of different behaviours occurring simultaneously; and the greater variety of ages in the capuchins (juveniles, sub-adults and adults) compared to the squirrel monkeys (mostly adults and sub-adults), again resulted in a higher probability of different behaviours occurring (e.g. juveniles more likely to play than adults).

In summary, using a novel modelling technique, we have contributed to the methodological approach to understanding ‘groupness' [13]. We found that capuchins and squirrel monkeys do not appear to synchronize their behaviour with other group members in captivity, suggesting that the environmental conditions (e.g. shared enclosures and husbandry) play a minor role in driving synchrony. By contrast, foraging advantages and reduced risk of predation, as predicted by the *double benefits hypothesis* [2], might provide more satisfactory explanations for the synchrony observed across MSGs in the wild. Although there is minimal risk of predation in captivity, future studies may want to consider investigating the effect of threats (i.e. through novel objects) on mixed-species synchrony in captivity. More broadly, integrating knowledge of species' behavioural ecology in the wild with studies of synchrony in captive settings will provide important insights for housing multiple species together in a single exhibit—especially those that are sympatric and form MSGs in the wild—which has become a common strategy in many zoos [46]. For instance, in addition to the *double benefits of grouping* [2], it is also important to consider the type of environment in which they live, for example, vertical stratification [47], and/or risk perception in relation to vegetation cover. Finally, understanding behavioural synchrony of animals can further aid in determining the treatment of each species in an exhibit, particularly if the behaviours may negatively impact the welfare of the animals. It is therefore necessary to take both environmental and social contexts into consideration in captive studies [32,46,48].

## Data Availability

The data are provided in electronic supplementary material [51].
